# A novel multi-epitope vaccine induces protective and therapeutic immunity against *Helicobacter pylori*

**DOI:** 10.1038/s41541-026-01409-9

**Published:** 2026-03-16

**Authors:** Hassan Moeini, Amrollah Mostafazadeh, Lars Schoenemann, Abbas Yadegar, Shaghayegh Jamshidizadeh, Kosar Nayeri, Volker Wedershoven, Florian Anderl, Christian Schulz, Bastian Popper, Peter Malfertheiner, Behnam Kalali

**Affiliations:** 1 Iguana Biotechnology GmbH, Munich, Germany; 2 Medical Clinic II, University Hospital of Munich, Ludwig Maximilian University Munich, Munich, Germany; 3 Cellular and Molecular Biology Research Center, Babol University of Medical Sciences, Babol, Iran; 4 Recombinant Protein Expression Facility, Rudolf-Virchow-Center, University of Wuerzburg, Wuerzburg, Germany; 5 Foodborne and Waterborne Diseases Research Center, Research Institute for Gastroenterology and Liver Diseases, Shahid Beheshti University of Medical Sciences, Tehran, Iran; 6 Department of Immunology and Microbiology, Babol University of Medical Sciences, Babol, Iran; 7 Core Facility Animal Models, Biomedical Center, Faculty of Medicine, Ludwig-Maximilian University, Munich, Germany

**Keywords:** Immunology, Microbiology

## Abstract

The development of an effective vaccine against *Helicobacter pylori* remains a major global health priority, aimed at reducing infection prevalence and preventing diseases such as chronic gastritis, peptic ulcers, and gastric cancer. Despite extensive research, no vaccine has yet demonstrated durable efficacy in clinical settings. This study describes the development of a novel multi-epitope vaccine targeting *H. pylori*. Sixteen B- and T-cell epitopes derived from six virulence factors were identified using bioinformatics tools and assembled into a Multi-Epitope Unit (MEU) antigen. The MEU antigen was formulated either as a flagellin-adjuvanted protein or delivered via a recombinant Modified Vaccinia virus Ankara (MVA) viral vector. Vaccine immunogenicity and efficacy were assessed in *H. pylori* SS1-challenged mice. Both formulations induced robust MEU-specific antibody and CD4^+^ T-cell responses, with the strongest immune responses observed following MEU-flagellin priming combined with MVA-MEU. The vaccines elicited balanced Th1/Th2 immunity and increased CD4^+^NKT-like cells frequencies. Notably, heterologous prime-boost vaccination or two doses of the MVA-MEU achieved complete bacterial clearance in both prophylactic and therapeutic models. These findings support the potential of MEU-based vaccines for preventing and treating *H. pylori* infection, thereby providing a strong rationale for advancement into toxicology studies clinical development.

## Introduction

Currently, no alternative therapies beyond antibiotic-based treatments effectively cure *Helicobacter pylori* infection, prevent gastritis and peptic ulcers, or mitigate the increased risk of gastric adenocarcinoma over time^1^. High antibiotic resistance reduces the efficacy of existing treatments and leads to rising rates of persistent infection^2^. Consequently, vaccine development remains a high-priority research topic^2,3^. However, efforts to develop such a vaccine have ultimately failed due to numerous challenges, including the bacterium’s complex biology, its ability to evade immune system attacks, and the high genetic diversity of strains^4–6^. Monovalent vaccines (containing only a single *H. pylori* antigen) have shown insufficient efficacy. In contrast, multivalent formulations targeting multiple antigens are expected to be more effective by eliciting stronger immune responses. Nevertheless, to date, candidates intended for prophylactic use that demonstrated potent efficacy in preclinical studies (primarily targeting conserved antigens such as CagA, VacA, HpaA, and various outer membrane proteins) have failed to translate into effective vaccines for human use. Despite eliciting a robust antibody response, recombinant antigen-based vaccines have failed to confer durable protection or sterile immunity in clinical trials^5,7–12^. Accumulating evidence now indicates that antibody titers alone do not correlate with long-term protection against *H. pylori*. Instead, durable immunity appears to depend on the induction of antigen-specific cellular immune responses, particularly CD4⁺ T-cell–mediated immunity and appropriate T-helper polarization. Current strategies focus on identifying the most conserved antigens, optimizing adjuvants, and improving delivery platforms^5,6^. The large molecular weight of *H. pylori* protein antigens hinders the development of multivalent vaccines. To overcome this obstacle, epitope-based vaccines (focusing on specific regions of antigens recognized by the immune system) may offer an innovative and promising solution^13,14^. By enabling precise targeting of immunodominant and conserved epitopes, such approaches have the potential to elicit broader, more durable immune responses while minimizing unnecessary antigenic load.

Our study explores the development of a novel multi-epitope *H. pylori* vaccine targeting B-cell and T-cell epitopes derived from six highly conserved virulence factors of the bacterium. These virulence factors include urease β-subunit (essential for *H. pylori* survival in acidic environments), FliD (a flagellar protein involved in bacterial motility), HpaA (a heparin-binding adhesion protein), HP0231 (a thiol-disulfide oxidoreductase involved in bacterial virulence), NapA (a neutrophil-activating protein that aids immune evasion), and BabA (an adhesion molecule critical for binding to host gastric epithelial cells). By combining broad epitope coverage with vaccination strategies designed to enhance cellular immunity and immune memory, this approach aims to address key limitations of earlier vaccine candidates. Taken together, these features provide a strong rationale for expecting improved and potentially more durable protective efficacy compared with previous antibody-focused vaccine strategies, thereby supporting the significant promise of this vaccine concept for effective prevention and treatment of *H. pylori* infection.

## Results

### Prediction of B-cell and T-cell epitopes

Through comprehensive bioinformatics analysis, sixteen B-cell and T-cell epitopes were carefully selected, as detailed in Table 1. For T-cell epitopes, both MHC classes I and II elements were considered.

**Table 1:** Predicted B-cell and T-cell epitopes from six *H. pylori* proteins

Protein	Location	Epitope Sequence^a^	Type of Epitope	Antigenicity Score
**Urease B**	46–61	GGGKTLREGMSQSNNP	B-cell	1.3452
	211–225	IEAGAIGFKIHEDWG	B-cell	0.9477
	229–251	SAINHALDVADKYDVQVAIHTDT	T-cell	N.A.
	349–384	TLHDMGIFSITSSDSQAMGRVGEVITRTWQTADKNK	B-cell	0.4515
**FliD**	55–64	TLLSSLKGPV	T-cell	N.A.
	107–129	QGDINELGAKFSSRDDIFSQVDT	B-cell	0.6162
	161–192	TNGEVMGIVMKTGGNDPYQLMVNTKNTGEDN	B/T-cells	1.1695
	570–600	SALNSNPKATQDFFYGSDSKDMGGREIHQEG	B/T-cells	0.9599
**HpaA**	12–32	ALKVEQILQNQGYKVISVDS	T-cell	N.A.
	61–98	DPKRTIQKKSEPGLLFSTGLDKMEGVLIPAGFIKVTIL	B/T-cells	0.5451
	99–147	EPMSGESLDSFTMDLSELDIQEKFLKTTHSSHSGGLVSTMVKGTDNSND	B/T-cells	0.8559
**HP0231**	25–61	SANDKRMQDNLVSVIEKQTNKKVRILEIKPLKSSQD	B/T-cells	0.8855
	179–197	RMVVVGWLGVNSAKKAALI	T-cell	N.A.
**NapA**	60–72	QLGHHPLVTLSEA	B-cell	0.5377
	76–86	TRVKEETKTSF	B-cell	0.8386
**BabA**	197–222	SAINTNEQSTPIGESGKNFNPFKDAS	B-cell	0.855

^a^Predicted T-cell epitopes within the amino acid sequences of mixed B-cell and T-cell epitopes are underlined.

### Design and construction of the multi-epitope unit-based vaccine

**Fig. 1:**
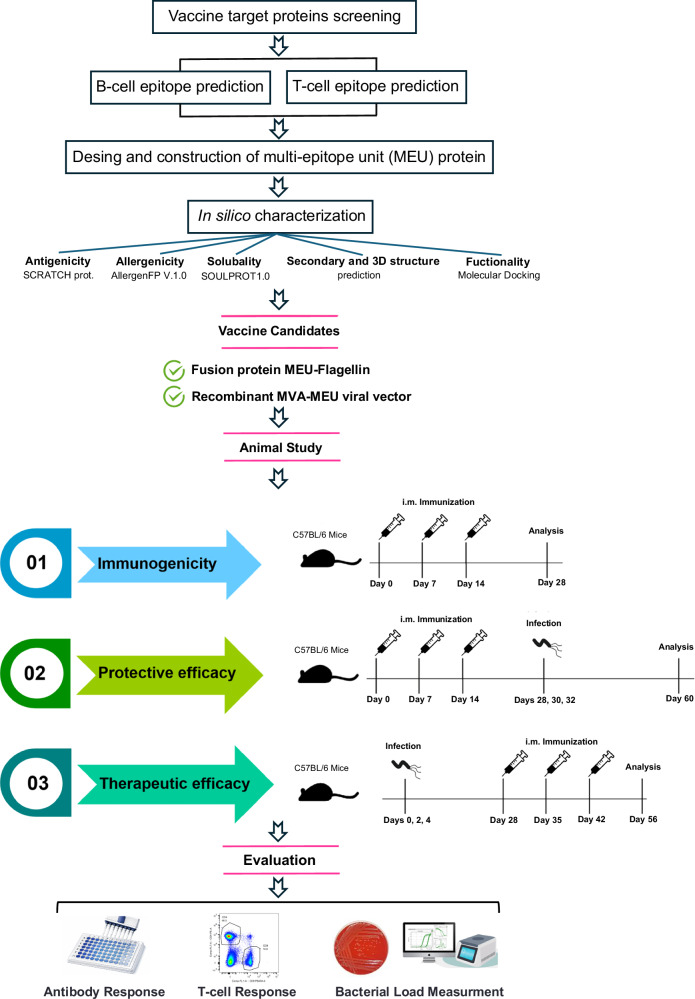
Schematic overview of the vaccine design and preclinical evaluation workflow. Target proteins were screened, and B- and T-cell epitopes were predicted to construct a multi-epitope unit (MEU). Following in silico characterization, selected candidates were developed as a fusion protein and a recombinant MVA vector and evaluated in mice for immunogenicity, protective efficacy, and therapeutic efficacy.

The predicted epitopes of six candidate bacterial proteins were tandemly connected in a manner that ensured the multi-epitope unit vaccine met the required standards for antigenicity, allergenicity, and physicochemical properties. The KK and GS linkers were used to promote epitope presentation and prevent the formation of neo-epitopes^14,15^. To enhance immune responses targeting MEU, *Salmonella* flagellin–renowned for its adjuvant properties–was incorporated into the construct. Specifically, the MEU antigen was fused with domains 0 (D0) and 1 (D1) of flagellin at its N- and C-terminal ends, respectively. Figure 1A shows a schematic representation of the MEU-Flagellin fusion protein. The fusion protein comprises 704 amino acid residues with a theoretical isoelectric point (pI) of 9.28, reflecting its fundamental physicochemical properties. The protein exhibited a predicted antigenicity probability of 100%, indicating strong potential to trigger an immune response. Additionally, the construct was predicted to be non-allergenic using Allergen FP V.1.0, with a solubility index of 0.56, suggesting its high likelihood of solubility.

The predicted secondary structure of the fusion protein (Fig. 1B) reveals its composition of α-helices, β-strands, and coiled regions, with the MEU moiety predominantly consisting of intertwined looped regions, as well as a few short helices and a 3-stranded β-sheet. The N- and C-terminal regions (corresponding to the flagellin D0 and D1 domains) are predominantly composed of α-helices, which are essential for maintaining the characteristic elongated structure of flagellin. These helical segments are interspersed with short, coiled regions, potentially contributing to structural flexibility. In contrast, the ventrally located MEU domain exhibits a more complex folding pattern characterized by combinations of α-helices, β-strands, and loops, distinguishing it from the flagellin arms. The 3D structural model (Fig. 1C) further supports this arrangement, where the flagellin arms adopt an extended α-helical conformation that transitions into a compact, globular MEU domain. This structural transition indicates a functional interplay between the two regions, which is critical for the protein’s overall activity. Although the MEU-flagellin fusion protein possesses relatively few secondary structural elements, the presence of these structural motifs contributes to its overall stability and supports its intended biological function.

### Interaction between fusion MEU-Flagellin protein and TLR5 receptor

Protein-protein docking analysis demonstrated a strong predicted interaction between TLR5 and the flagellin D1 domain within the MEU-flagellin fusion protein, with favorable SwarmDock total energy score (E-total: −30.53 kJ/mol), indicating high affinity (Supplementary Fig. S1). Furthermore, molecular-docking results predicted that the TLR5 agonists within the fusion protein construct could efficiently bind to their receptor, similar to their natural counterparts, with an E-total of −38.12 for the TLR5-natural flagellin complex. The analysis of hydrogen bonds and hydrophobic interactions between the MEU fusion protein and TLR5 also indicated a proper interaction between the TLR5 and the D1N domain of flagellin in the fusion protein, analogous to the interactions observed with the natural ligand.

### Expression, purification, and characterization of the MEU-Flagellin fusion protein

The MEU-flagellin fusion protein was successfully expressed in *E. coli* BL21 (DE3), predominantly existing as inclusion bodies. The purification process involved sequential washing of the inclusion bodies with Triton X-100 and NaCl. The inclusion bodies were then solubilized using 8.0 M urea. Following the two rounds of purification via ion exchange chromatography using a HiTrap SP FF column (Supplementary Fig. S2A), the eluted fractions containing the fusion protein were pooled, concentrated, and subjected to size exclusion chromatography (SEC) for final purification. SDS-PAGE analysis confirmed the successful purification of the MEU-flagellin fusion protein, exhibiting a molecular weight of approximately 75.5 KDa (Supplementary Fig. S2B).

### TLR5 activation by MEU-Flagellin validates the adjuvant potential of the flagellin component

To assess the ability of the flagellin arms within the MEU protein to activate TLR5 signaling, a SEAP reporter assay was conducted using HEK-Blue hTLR5 cells. The results are summarized in Supplementary Fig. S2C. As expected, the positive control recombinant flagellin exhibited a strong induction of SEAP activity, confirming efficient TLR5 activation. In contrast, MEU alone failed to induce the absence of significant SEAP activity across all tested concentrations (10-640 pg/ml), indicating no TLR5 activation. However, the MEU-Flagellin fusion protein exhibited a dose-dependent increase in SEAP activity, reaching a plateau at 320–640 ng/ml, suggesting saturation of TLR5 activation. This demonstrates that the flagellin arms within the fusion protein retain their functionality as an effective adjuvant, capable of triggering TLR5-mediated signaling and enhancing immune activation.

### Construction of Recombinant MVA Encoding MEU Antigen

**Fig. 2:**
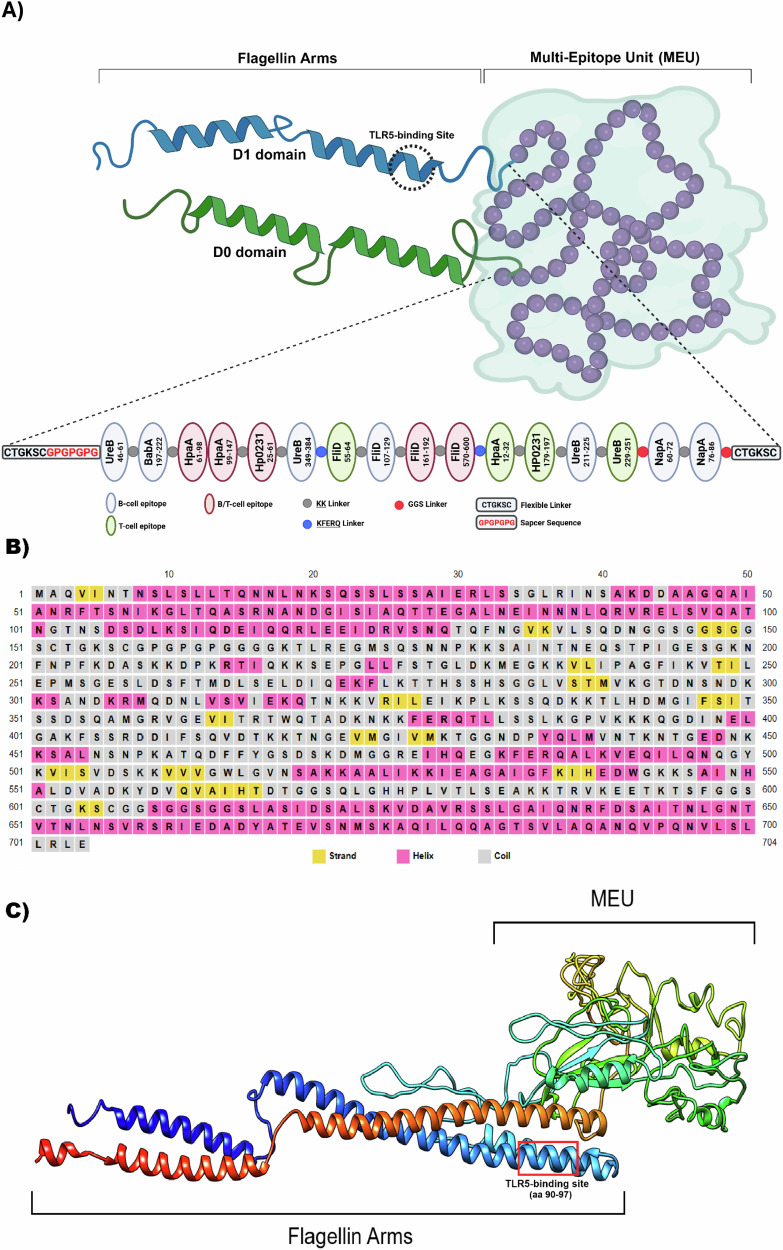
Structural design and modeling of the MEU–Flagellin fusion protein. **A** Schematic representation of the MEU–Flagellin construct comprising sixteen predicted B- and T-cell epitopes derived from six bacterial proteins. The N- and C-terminal domains of Salmonella flagellin were fused to the respective termini of the MEU sequence. **B** Predicted secondary structure showing predominantly α-helical flagellin N- and C-terminal domains and a structurally diverse MEU region composed of loops, short helices, and β-strands. **C** Three-dimensional structural model illustrating elongated α-helical flagellin arms transitioning into a compact, folded MEU domain.

**Fig. 3:**
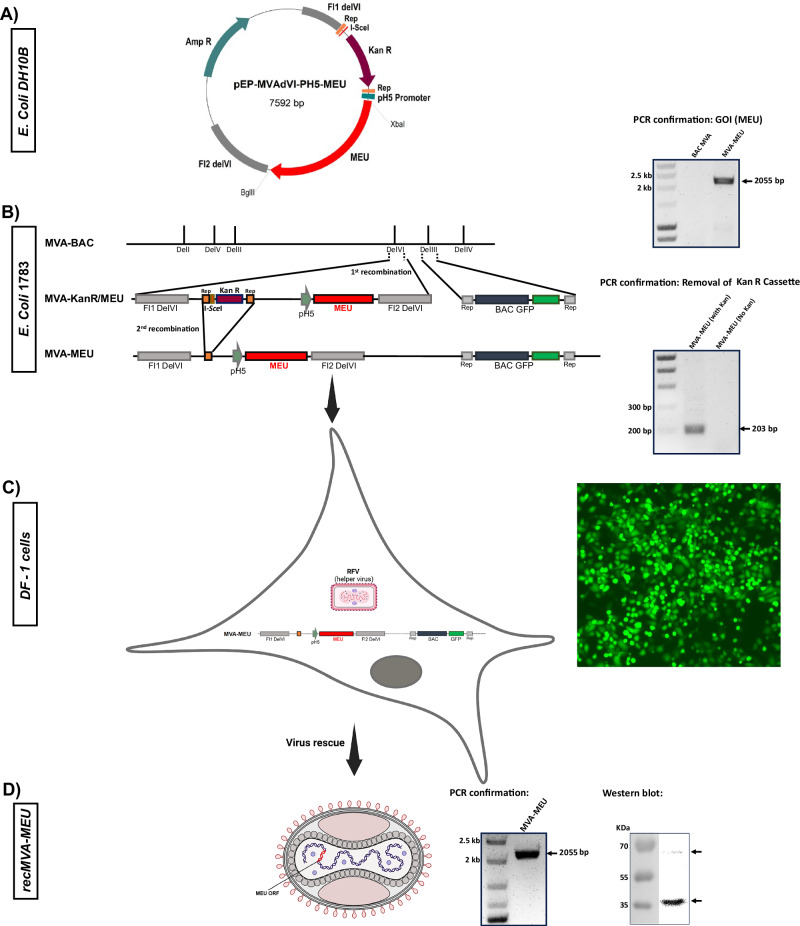
Generation and Confirmation of Recombinant MVA-MEU. **A** Schematic representation of the recombinant shuttle vector construction. The MEU gene fragment was inserted into the pEPMVAdVI PH5 plasmid downstream of the *PH5* promoter. **B** *Generation of recombinant MVA-BAC in E. coli 1783 via a two-step recombination process.* PCR amplification verified the successful insertion of the *I-SecI-Kan-pH5/MEU-Flag* fragment into the MVA genome of the BAC plasmid. Following arabinose-induced excision of the *KanS* cassette and a second recombination event at 42 °C, the correct clones were identified by PCR using *DelVI*-specific primers. **C** Rescue and propagation of recMVA-MEU. Recombinant viruses were rescued in DF-1 cells in the presence of RFV helper virus and recMVA-BAC plasmid. GFP expression, observed under fluorescence microscopy, confirmed expression of the BAC plasmid carrying the eGFP gene (Right panel). **D** Characterization of rescued viruses. PCR verified the presence of the transgene in the viral genome (Right panel). Western blot analysis detected distinct MEU bands in infected DF-1 cells (Right panel).

A recombinant shuttle vector carrying the MEU gene fragment was constructed by inserting the MEU coding sequence into the *Bgl*II and *Xba*I sites of the pEPMVAdVI PH5 plasmid downstream of the PmH5 promoter (Fig. 2A). Following transformation into *E. coli* competent cells, correct clones were identified via restriction enzyme digestion and PCR using gene-specific and *Del*VI primers listed in Table 2. Potentially correct clones were sequenced to verify integrity of the inserted fragments. To construct the recombinant MVA-BAC, the transgene cassette, along with the Kan cassette, *I-Sec*I sequence, and *Del*VI flanking sequences, were amplified from the shuttle vectors and introduced into the GS1783 *E. coli* competent cells carrying the MVA/BAC. As shown in Fig. 2B, PCR amplification confirmed insertion of the *I*-*Sec*I-Kan-pH 5/MEU-Flag fragment into the MVA genome in the BAC plasmid. The KanS-cassette was removed by arabinose-induced endonuclease I-*Sce*I, followed by a second red recombination at 42 °C in GS1783 *E. coli* cells. Correct clones were confirmed by PCR using specific *Del*VI primers (Fig. 3B). The recMVA-BAC plasmid was transfected into DF-1 cells after infection of the cells with the helper virus RFV (Fig. 2C). After 2–3 days of incubation at 37 °C, cells were harvested, and the recombinant viruses were used to infect new DF-1 cells. The BAC plasmid carrying the eGFP gene enabled the screening of successful recMVAs through GFP expression, observable under fluorescence microscopy. PCR analysis revealed the presence of the transgene in the viral genome (Fig. 2C, Left side). Subsequently, Western blot analysis was performed to detect the expression of the heterologous MEU gene in recMVA-MEU (Fig. 3D, Left side). The viral particles were then concentrated using a 36% sucrose cushion, yielding a virus titer of 2×10^10^ viral particles/ml, based on the TCID50 assay.

**Table 2:** List of primers used in this study

Primer	Sequence (5′ → 3′)	Reference
Kan control	F: CGTACTCCTGATGATGCATG R: ATTCGTGATTGCGCCTGAGC	^34^
MVA *Del*VI	F: CTCCGCATCTAGTTGATATTCCAACCTCTT R: CCTGGACATTTAGTTTGAGTGTTCCTGAAT	^34^
pEP-MEU	F: TAAGGTTGACTCTAGAGCCACCATGTGCACTGGAAAGTCCTGTGG R: ACGTAGAGCTCTTAAGAGATCTTTAGCATGATTTGCCGGTACAAC	This study
REV control	F: AAAGATGCGTACATTGGACCC R: GTTCGAGACTAGAAAAGCGCC	^34^
qHP-FliD	F: AGAGCAACACCAGCGAAGAAA R: GCCTTCGGCGTTCTTAAACG	This study
HP-glm	F: GGATAAGCTTTTAGGGGTGTTAGGGG R: GCTTACTTTCTAACACTAACGCGC	^35^

### MEU antigen induces a significant level of antibody and T-cell responses in mice

**Fig. 4:**
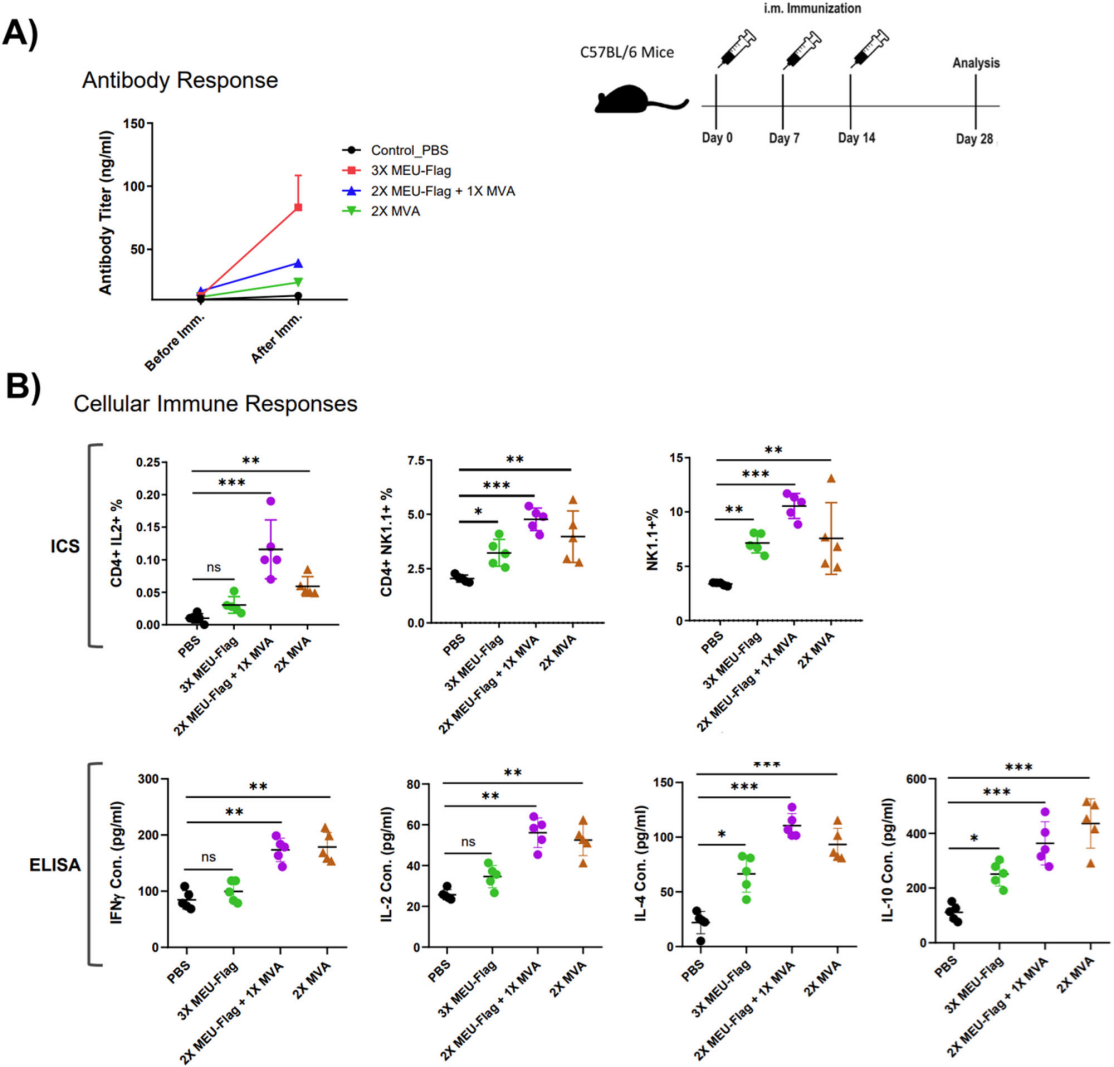
Evaluation of humoral and cellular immune responses in vaccinated mice. **A** Assessment of antibody responses by measuring antigen-specific IgG levels in serum via ELISA. **B** Analysis of cell-mediated immune responses using intracellular cytokine staining (ICS) to quantify cytokine-producing T cells and ELISA for determining cytokine secretion profiles from stimulated splenocytes. Data represent mean ± SEM from 5 independent experiments.

The immunogenic potential of the MEU-based vaccine was assessed in C57BL/6 mice immunized intramuscularly with MEU alone, flagellin-adjuvanted MEU, the recombinant MVA viral vector encoding MEU administrated alone, or in combination with the MEU-based protein using a prime-boost vaccination strategy. This involved assessing the induction of MEU-specific antibody and T-cell responses in immunized mice. Antibody titers were evaluated on days 0, 7, and 14 post-immunizations, as shown in Fig. 4A. By day 14, antibody titers in both the FusionMEU+MVA and FusionMEU+MVA+Boost groups were significantly higher than in the control group. Notably, the FusionMEU+MVA+Boost group elicited the highest antibody titers, highlighting the enhanced immunogenicity provided by the additional booster dose. Mice immunized with two doses of recMVA showed a slight increase in antibody titers, but the effect was less pronounced than with the FusionMEU-based protocols.

To further elucidate the cellular immune responses elicited by MEU-based vaccine formulations, T-cell-related immune parameters were analysed via intracellular cytokine staining on mice splenocytes two weeks post-final immunization. This assay compared cytokine profiles among naive mice, mice immunized with the homologous MEU-based vaccine candidate, and mice receiving a heterologous prime-boost immunization strategy. Results shown in Fig. 4B revealed a significant increase in CD4^+^ T cells producing IL-2 in mice vaccinated with either the Flag-MEU or the combined Flag-MEU + MVA strategy compared to the PBS control group. Notably, the Flag-MEU + MVA regimen demonstrated the highest percentage of CD4^+^IL-2^+^ T cells, indicating a robust antigen-specific response. Additionally, the vaccine formulations led to an increased level of CD4 + NKT-like cells, a key immune subset that bridges innate and adaptive immunity. The Flag-MEU + MVA approach induced a significantly higher frequency of CD4 + NKT-like cells compared to the individual immunization strategies, underscoring its enhanced immunogenicity. Notably, elevated levels of secreted IFN-γ and IL-2 were detected in the supernatant of splenocyte cultures stimulated with MEU-pooled peptides, indicating Th1 activation. Concurrently, increased production of IL-4 and IL-10 suggested the involvement of Th2 pathways. This dual activation highlights the potential of the MEU-based vaccine candidate to elicit a balanced immune response, with Th1 pathways contributing to pathogen clearance and Th2 pathways supporting humoral immunity. Such a comprehensive immune response underscores the promise of the MEU-based strategy in combating *H. pylori* infections.

### Recombinant MVA, alone or in combination with Flagellin-adjuvanted MEU in a heterologous prime-boost immunization strategy, protects mice against H. pylori infection

To evaluate the potential of MEU-based vaccine candidates against bacterial infection, mice were challenged with live *H. pylori* SS1 strain two weeks after the final immunization. Four weeks later, bacterial load in the stomach was assessed via PCR and culture, with results compared to those of unimmunized control mice. As shown in Fig. 3A, both vaccine protocols, using the flagellin-adjuvanted MEU and recMVA, demonstrated complete protection against *H. pylori* infection compared to unimmunized animals. This protection was achieved through either a schedule of immunization with the protein-based vaccine candidate followed by an MVA booster, or through two administered doses of MVA.

### MEU-based vaccine eradicates established H. pylori Infection in Mice

The therapeutic potential of MEU-based vaccine candidates against chronic *H. pylori* infection was assessed in C57BL/6 mice, in which mice were first infected with the live *H. pylori* SS1 strain to establish chronic infection. Subsequently, immunization was carried out using the MEU-based vaccine delivered either as a flagellin-adjuvanted protein formulation or within the MVA vector. The immunization schedule comprised two doses of FusionMEU or MVA, administered either as a homologous or heterologous prime-boost strategy, with a booster dose administered where applicable. As shown in Fig. 3B, the FusionMEU prime + MVA boost regimen induced the robust antigen-specific IgG responses, whereas 2× MVA vaccination alone resulted in a modest increase in IgG titers, which was higher than control groups but substantially lower than the heterologous prime–boost regimen. Subclass distribution of serum IgG antibodies was further analysed to determine whether the vaccine candidates influenced the distribution of IgG subtypes. The ratio of antigen-specific IgG1 to IgG2a antibodies, along with cytokine profiling, characterizes T-helper (Th) cell bias, with IgG1 and IgG2a serving as markers for Th2 and Th1 lymphocytes, respectively. A ratio between 0.5 and 2.0 signifies a mixed or balanced Th1/Th2 response, while ratios below 0.5 suggest a Th1-polarized response and ratios above 2.0 indicate a Th2-polarized response. This classification reflects the role of Th1 responses in cell-mediated immunity and the role of Th2 responses in humoral immunity. As depicted in Fig. 3B, the ratios of MEU-specific IgG1:IgG2a following intramuscular immunization with MEU antigen alone or in combination with flagellin, or as part of a prime-boost vaccination regimen in mice boosted with recMVA-MEU, demonstrated a balanced Th1/Th2 response with ratios ranging from 0.5 to 2.0. These findings indicate that the immune response elicited by the MEU-based vaccine is neither strongly Th1-dominant nor Th2-dominant but rather represents a mixed polarization supporting both cell-mediated and humoral immunity.

The observed humoral responses in vaccinated mice were coupled with cellular immune responses, as demonstrated by elevated levels of NKT cells. Specifically, the FusionMEU + MVA group displayed increased levels of CD4 + NKT-like cells, whereas the MVA (2×) group showed increased levels of CD8^+^ NKT-like cells. Therapeutic efficacy was evaluated by quantifying bacterial loads in stomach tissues three weeks after the final immunization. Quantitative culture and PCR analysis (Fig. 3B) demonstrated complete bacterial clearance in both vaccination strategies. In contrast, untreated control animals maintained high bacterial loads. These findings indicate that MEU-based vaccines can eradicate established *H. pylori* infections in mice, highlighting their potential as an effective therapy for chronic *H. pylori* infections. The combination of robust antibody responses, targeted NKT-cell prevalence, and significant bacterial clearance underscores the potential of MEU-based vaccines as a therapeutic intervention for chronic *H. pylori* infections.

## Discussion

In this study, we developed a novel multi-epitope vaccine targeting *H. pylori*, formulated either in a fusion protein with flagellin or incorporated into a recombinant MVA viral vector. This vaccine candidate was intentionally designed to overcome key limitations associated with previously evaluated subunit vaccines against *H. pylori*. Rather than relying on full-length antigens, our approach focuses on conserved B-cell and T-cell epitopes derived from six distinct virulence factors, with the aim of eliciting robust and targeted humoral and cellular immune responses. A major challenge in *H. pylori* vaccine development has been that several vaccine candidates elicited robust systemic antibody responses yet failed to confer durable protection in humans. Clinical studies with recombinant vaccines incorporating antigens such as CagA, VacA, and neutrophil-activating protein demonstrated high antigen-specific IgG titres and measurable T-cell responses in vaccinated individuals; however, these immune responses did not translate into protection following challenge with virulent *H. pylori* strains^12^. Similarly, subunit vaccines based on urease administered with conventional mucosal adjuvants generated detectable antibody responses but showed limited or inconsistent efficacy in clinical settings^11^. This translational gap between immunogenicity and protection highlights the importance of vaccine strategies that induce balanced humoral and cellular immunity, which are likely critical for the control and clearance of chronic gastric infection.

To promote coordinated humoral and cellular immune responses, T-cell epitopes were derived from B-cell epitope-containing regions. This approach aligns with stimulating a comprehensive immune response, which is necessary for effectively clearing persistent pathogens like *H. pylori*. To enhance immunogenicity and structural coherence, epitopes were linked using KK and GS linkers. These linkers are widely recognized for preserving the native conformation of individual epitopes, improving epitope presentation, and reducing the risk of neo-epitope formation^14,15^. The final construct, termed the multi-epitope unit (MEU), was designed to be both immunogenic and biochemically stable. To further augment the immune response, Salmonella flagellin domains D0 and D1 were incorporated as a potent immunostimulatory molecule that activates innate immunity via TLR5 signaling^16,17^ at the N- and C-termini of the MEU construct, respectively. The D1 domain directly interacts with TLR5, while D0 maintains structural integrity to ensure proper folding of the flagellin arms^18–21^. This configuration ensures that the vaccine construct retains its adjuvant potential while maintaining antigenic specificity. The high conservation of D0 and D1 across bacterial species further reduces the risk of eliciting off-target immune responses.

Physicochemical analysis confirmed that the MEU-Flagellin fusion protein is stable, non-allergenic, and highly antigenic. The strong antigenicity and non-allergenicity of this construct support its potential to elicit a robust and effective immune response with minimal risk of adverse effects. Secondary structure analysis revealed that the flagellin domains were predominantly alpha-helical, consistent with their known structural characteristics, whereas the MEU domain exhibited a mixture of alpha-helices, beta-sheets, and loops, indicating a well-folded and stable core. Tertiary structure modeling corroborated these findings, showing a compact globular MEU domain flanked by extended helical flagellin arms.

Molecular-docking analysis was employed to evaluate the interaction between the MEU-Flagellin fusion protein and TLR5, revealing strong binding affinity between the D1 domain and TLR5. This affinity is comparable to that of native flagellin, which suggests that the fusion protein retains the functional capabilities of natural TLR5 ligands. This finding is significant because proper structural and functional interactions between TLR5 and the fusion protein are essential for effective immune activation. To validate the in-silico predictions, a SEAP reporter assay was performed using HEK-Blue hTLR5 cells. This assay demonstrated that the MEU-Flagellin fusion protein induced a dose-dependent increase in SEAP activity, confirming the functional activation of TLR5. In contrast, the MEU construct without flagellin failed to stimulate SEAP activity, underscoring the essential role of the flagellin component in TLR5-mediated signaling. The plateau in SEAP activity at higher concentrations indicates that the fusion protein reaches an optimal threshold for receptor activation, a desirable feature for vaccine adjuvants.

In parallel with the MEU-Flagellin fusion construct, the MEU antigen was also incorporated into a recombinant Modified Vaccinia Ankara (MVA) viral vector. MVA is a well-characterized vaccine platform known for its ability to enhance T-cell responses, particularly in heterologous prime-boost strategies^22,23^. The MVA-MEU vector was developed to evaluate its potential both as a standalone vaccine and as a booster when combined with the protein-based formulation. The MVA vector adds a valuable layer of immunostimulation by presenting the MEU antigen in a highly immunogenic viral context.

Immunogenicity studies in C57BL/6 mice demonstrated that MEU-based vaccination induced robust antigen-specific immune responses. Compared with MEU alone, both MEU-flagellin and MEU + MVA regimens elicited significantly higher MEU-specific IgG titers, confirming the immunostimulatory contribution of flagellin and the boosting capacity of MVA. However, consistent with emerging evidence from both murine and human studies, including recent clinical and translational reports, antibody titers alone did not fully account for protection against *H. pylori*, highlighting the importance of cellular immune responses^24^.

To further characterize the quality of the humoral response and its association with underlying T-helper polarization, we analysed IgG subclass distribution, The results showed balanced Th1/Th2 responses in MEU-based vaccine groups, with IgG1:IgG2a ratios ranging from 0.5 to 2.0. This balance is particularly important in *H. pylori* infection, as Th1 responses contribute to bacterial clearance while Th2 responses promote antibody production and help mitigate inflammatory damage^25^. Cytokine profiling supported this finding, revealing elevated levels of the Th1-associated cytokines IFN-γ and IL-2, alongside increased levels of the Th2-asspciated cytokines IL-4, and IL-10. Further characterization of cellular responses revealed significant activation of CD4 + T cells, particularly Il-2-producing sunsets, with the strongest responses observed in the MEU + MVA group. These findings are consistent with recent reports identifying CD4 + T-cell–mediated immunity as a key correlate of protection against *H. pylori*^24,26^. Additionally, elevated levels of NK- and CD4 + NK-like T-cells were observed in vaccinated mice, highlighting their instrumental role in bridging innate and adaptive immunity^27^. The absence of excessive CD8 + T-cell activation minimizes the risk of cytotoxic tissue damage, a concern in *H. pylori*-infected gastric mucosa^25^.

MEU-based vaccines demonstrated robust efficacy in both prophylactic and therapeutic settings of *H. pylori* infection, achieving complete bacterial clearance as measured by PCR and bacterial culture. The ability to both prevent infection and eradicate established infection underscores the strength of selected epitopes and the vaccination strategies employed. Previous studies have provided evidence that multi-epitope vaccine designs can induce protective immune responses by targeting multiple T-cell epitopes, supporting the conceptual validity of this approach^28^. The dual prophylactic and therapeutic efficacy observed in our study is likely attributable to the breadth antigenic coverage achieved by incorporating epitopes from six *H. pylori* virulence factors together with the complementary use of protein-based vaccination and viral vector–based delivery system. Unlike earlier vaccine candidates that focused on limited antigens^4–6,9,12^, our construct targets multiple epitopes from six virulence factors, thereby enhancing immune breath and potentially limiting immune evasion.

The inclusion of MVA as a viral vector booster further augmented the vaccine’s efficacy. Beyond enhancing CD4 + T-cell responses, MVA significantly increased the level of CD4 + NK1.1+ cells, a subset critical for early immune engagement and long-term protection. These cells play a central role in coordinating immune responses by activating other lymphocyte subsets and promoting immunological memory^27,29^. Our findings indicate that the MEU + MVA prime-boost regimen effectively leverages these mechanisms. The induction of such cellular immune profiles closely mirrors correlates of protection identified in recent human vaccination studies^24^, strengthening the translational relevance of our findings. Notably, the absence of a robust CD8^+^ T-cell response, which could otherwise induce unwanted inflammatory effects at the site of infection, further supports the vaccine’s safety profile. Previous research has shown that exaggerated CD8^+^ T-cell responses can exacerbate tissue damage in the gastric mucosa^25^.

From a translational research perspective, the concurrent use of a protein subunit and a viral vector provides flexibility in vaccine development and optimization. In the context of the present study, MEU-based vaccines achieved complete bacterial clearance in both prophylactic and therapeutic mouse models. These results support the potential of the selected epitopes and vaccination strategies under the experimental conditions used. However, further studies will be required to evaluate long-term immunity, cross-protection against diverse *H. pylori* strains, and optimal dosing regimens before clinical translation can be considered.

In conclusion, the results of this study confirm the significant potential of the MEU-based vaccine candidates against *H. pylori* infection. By integrating epitope-based antigen design with a potent adjuvant (flagellin) and an MVA-based viral vector, we elicited robust humoral and cellular immune responses with a balanced Th1/Th2 profile. This strategy conferred sterilizing protection against *H. pylori* challenge, achieved full bacterial clearance of established infections in our model, and demonstrated a favorable safety profile.

Ongoing work includes toxicology studies in rats and preparations for the first-in-human trials. If successful, the vaccine holds promise to reduce the global burden of *H. pylori*-associated diseases and help combat antimicrobial resistance.

## Methods

### Bacterial virulent factors sequence analysis

**Fig. 5:**
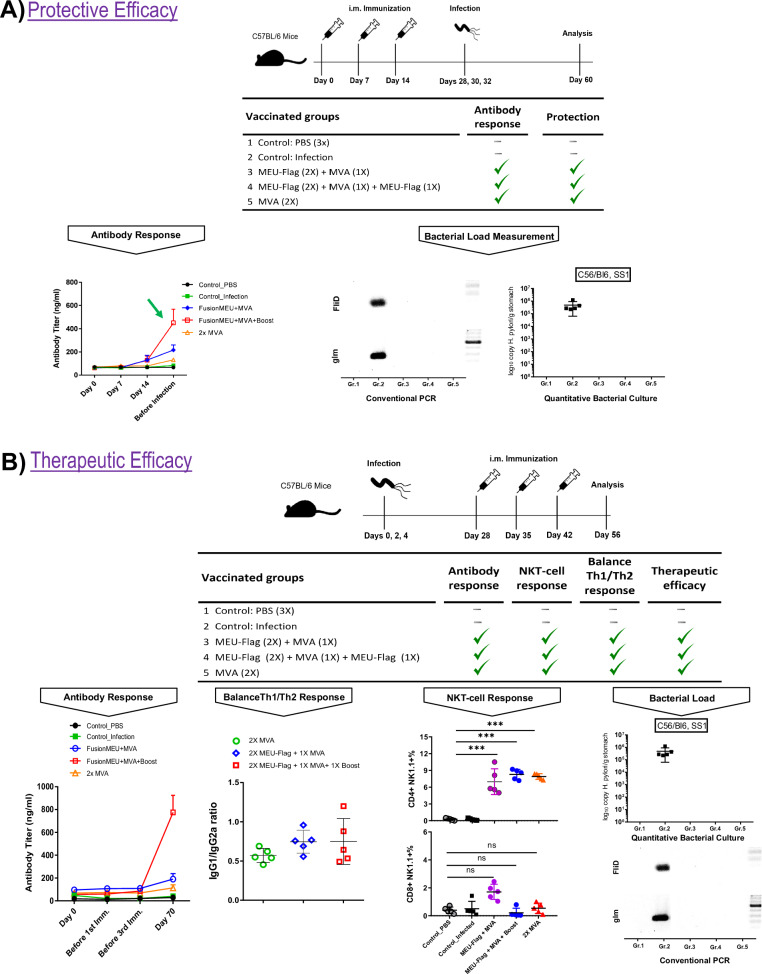
Assessment of the protective and therapeutic efficacy of the vaccine. **A** *Protective efficacy***:** C57BL/6 mice were immunized intramuscularly on days 0, 7, and 14 using homologous or heterologous prime–boost regimens, as indicated. In heterologous groups, mice were primed with the MEU-Flagellin protein and boosted with the recombinant MVA-MEU vector. Mice were challenged with *H. pylori* SS1 on days 28, 30, and 32, and analyses were performed on day 60. Vaccine-induced protection was evaluated by measuring antigen-specific antibody responses via ELISA, quantifying bacterial load through PCR targeting the *FliD* and *glmM* genes, and assessing bacterial burden via quantitative bacterial culture. **B** *Therapeutic efficacy***:** C57BL/6 mice were first infected with *H. pylori* SS1 on days 0, 2, and 4. Following establishment of infection, mice were immunized intramuscularly on days 28, 35, and 42 using homologous or heterologous prime–boost regimens, as indicated. In heterologous groups, vaccination consisted of priming with the MEU-Flagellin protein followed by boosting with the recombinant MVA-MEU vector. Immune responses and bacterial burden were analysed on day 56. Data represent mean ± SEM from 5 independent experiments.

This study selected six bacterial viral factors, including urease β-subunit (WP_000724295.1), FliD (Q9ZL91.3), HpaA (Q9ZL47.1), HP0231 (KT932668.1), NapA (A0A1U9ISG7_HELPX), and BabA (Q9ZKV2_HELP), for vaccine design against *H. pylori* infection. The research methodology of this study is depicted in Fig. 5.

### B-cell and T-cell epitope prediction

Linear B-cell epitopes for candidate bacterial proteins were predicted using the Immune Epitope Database (IEDB) (http://tools.iedb.org/main/bcell/) and the BepiPred epitope prediction tool (Department of Bioinformatics and Health Informatics, Technical University of Denmark, Copenhagen, Denmark). The antigenicity index of predicted epitopes was evaluated using VaxiJen v2.0 (Oxford University, Oxford, UK). T-cell epitopes within proteins were also predicted via the IEDB T-cell epitope prediction tool (http://tools.iedb.org/main/tcell/). Based on epitope prediction results, dominant epitopes with higher prediction rates and scores were selected for each protein.

### Construction of multi-epitope unit antigens

To construct the multi-epitope unit (MEU)-based vaccine, selected B-cell and T-cell epitopes were tandemly linked via either lysine-lysine (KK), a target cleavage site for lysosomal proteases, or glycine-serine (GS) linkers. The arrangement that met all predicted criteria regarding antigenicity, allergenicity, and physicochemical properties was chosen for constructing the multi-epitope unit vaccine. Additionally, the N-terminal (D1 domain: 142 AA) and C-terminal (D0 domain: 89 AA) domains from *Salmonella* flagellin were incorporated into the N- and C-termini of the MEU, respectively, serving as a TLR5-stimulating adjuvant to boost the immune response against the target MEU antigen. The physicochemical features of the fusion protein, including the theoretical isoelectric point (pI), were evaluated using the ProtPi online tool (https://www.protpi.ch/Calculator/ProteinTool).

### Profiling of MEU-Flagellin antigenicity, solubility, and allergenicity

The antigenicity of the MEU protein was evaluated using VaxiJen online tool(http://www.ddg-pharmfac.net/vaxijen/VaxiJen/VaxiJen.html)^30^, which predicts antigenic potential based on physicochemical characteristics such as hydrophobic amino acids, molecular weight, and polarity, independent of sequence alignment. A score above the threshold of 0.4 indicates potential antigenicity. Solubility was assessed using the SoluProt webserver (https://loschmidt.chemi.muni.cz/soluprot/)^31^, which predicts the likelihood of fusion proteins being expressed as soluble proteins in *Escherichia coli* (*E. coli*). Allergenicity was predicted using the AllerTOP server (http://www.ddg-pharmfac.net/AllerTOP/index.html)^32^, which was trained on allergen and non-allergen datasets to assess the allergenic potential of MEU antigens.

### Prediction of secondary and 3D structure of the MEU-flagellin antigen

The secondary structure of the MEU-Flagellin was predicted using the high-accuracy prediction tool PSIPRED (Protein Bioinformatics Group, University of Warwick, Coventry, UK). 3D models of the MEU-flagellin were generated using the I-TASSER 5.0 server^33^. Protein models were visualized using PyMOL software (Schrödinger Inc., New York, NY, USA).

### Molecular docking of MEU-Flagellin protein with TLR5

Flagellin is considered a potent pathogen-associated molecular pattern (PAMP) due to its ability to bind and activate Toll-like receptor 5 (TLR5), thereby triggering innate immune responses^18,19^. To investigate the molecular details of the interactions between the engineered Flagellin-MEU fusion antigen and TLR5, molecular docking analyses were performed between the predicted 3D structure of the Flagellin-MEU protein and the crystal structure of TLR5 (PDB: 3J0A) using the HDOCK server (http://hdock.phys.hust.edu.cn/). The molecular interactions of the best-docked complexes, identified through a molecular docking study, were analyzed using Dimplot in LigPlot (v.2.2) software. This program generates detailed schematic representation, highlighting hydrophobic interactions and hydrogen bonding patterns between receptors and ligands. The PDB file for TLR5 was obtained from the Protein Data Bank (https://www.rcsb.org/).

### In vitro TLR5 activation by flagellin components in the MEU protein

To evaluate the adjuvant function of flagellin arms in the MEU protein in vitro, the HEK-Blue-Luci hTLR5 cells (InvivoGen, France) were employed, expressing the human TLR5 receptor and an inducible secretory embryonic alkaline phosphatase (SEAP) under an NF-κB-inducible promoter. This approach quantifies TLR5 activation by measuring SEAP activity, offering a reliable platform for assessing the adjuvant potential of flagellin. For this purpose, HEK-Blue-Luci hTLR5 cells were seeded at a density of 2×10^4^ cells/well in a 96-well plate using DMEM medium supplemented with 10% FBS, 1% penicillin-streptomycin, and selective antibiotics, and allowed to adhere overnight. Subsequently, cells were treated for 24 h with serial dilutions of MEU-Flagellin fusion protein at concentrations of 10, 20, 40, 80, 160, 320, and 640 ng/ml, along with MEU alone (negative control) and recombinant flagellin (positive control: Invivogen, France), to activate TLR5 and thereafter initiate NF-κB signaling. After incubation, cell culture supernatants were collected and SEAP activity was quantified using QUANTI-Blue™ (a SEAP detection reagent, Invivogen, France). For the assay, 20 µl of cell culture supernatant was incubated with 180 µl of SEAP detection reagent at 37 °C for 20 min. The enzymatic conversion of the substrate resulted in a color change, which was measured at 620 nm using an ELISA reader (Tecan, USA). The optical density (OD) values were normalized to untreated controls.

### Recombinant MEU protein production and purification

The gene fragment encoding MEU-Flagellin was codon-optimized for efficient expression in *E. coli* and synthesized by Eurofins Genomics (Germany). The synthesized coding sequence was cloned into the pET-28b expression vector downstream of the T7 promoter. To ensure accuracy, the clones were screened by restriction enzyme digestion, and the correct orientation and sequence of the inserted fragment were verified by double-strand sequencing. The recombinant plasmid pET-28b/MEU-Flag was transformed into *E. coli* BL21 (DE3) as the host strain for expressing the MEU-Flagellin fusion protein. For this purpose, the transformed bacterial cells were cultured in 2 L of LB medium at 37 °C and 200 rpm. Protein expression was induced by adding 1 mM isopropyl β-D-1-thiogalactopyranoside (IPTG) when the OD_600_ reached 0.6, followed by overnight incubation at 18 °C. The bacterial cells were then harvested by centrifugation, and the pellet was resuspended in 84 mL of 50 mM Tris-HCl (pH 8.0). Subsequently, bacterial cells were lysed by sonication (4 cycles, 30 s each) and the lysate was cleared by centrifugation at 20,000 × *g* for 30 min. The resulting pellet was then subjected to sequential washing steps using 50 mM Tris-HCl (pH 8.0) containing either 1% (v/v) Triton X-100 (two washes) or 1 mM NaCl (one wash), followed by a final wash with H_2_O to obtain purified inclusion bodies. The final pellet was resuspended in 8.4 mL of H_2_O to yield 10 mL of inclusion bodies, which were directly used for solubilization. For solubilization, the inclusion body solution was diluted 1:10 in a solubilization buffer (8.8 M urea, 20 mM HEPES, pH 8.0) and stirred overnight at room temperature. Subsequently, the solution was cleared by centrifugation at 20,000 × *g* for 15 min and loaded onto a HiTrap SP FF 5 mL column equilibrated with IEX-A buffer (8 M urea, 20 mM HEPES, pH 8.0). After washing with 25 mL of IEX-A buffer, proteins were eluted using a step gradient of 100% IEX-B buffer (1x PBS, 250 mM L-Arginine) over 25 mL, with 2 mL fractions collected. Elution fractions containing the target protein, as determined by SDS-PAGE analysis in two IEX pilot runs, were pooled and concentrated to approximately 1 mL, yielding a final concentration of 2.28 mg/mL. The concentrated protein was then further purified using size exclusion chromatography (SEC) on a Superdex 200 10/300 Increase column pre-equilibrated with SEC buffer. A volume of 500 μL from the concentrated IEX pilot 2 pool was loaded onto the column, and fractions of 250 μL were collected. The purity of the eluted protein was assessed by SDS-PAGE analysis.

### Construction of recombinant MVA vector encoding the MEU antigen

First, the MEU gene fragment was amplified from the pEXK4-MEU-Flagellin plasmid using a pair of specific primers shown in Table 2. This fragment was then inserted into the *Xba*I and *Bgl*II sites of the shuttle vector pEP-MVAdVI-pH5 using the In-Fusion cloning kit. Following the transformation of *E. coli* competent cells, positive clones were screened on LB-Amp agar plates; plasmids were extracted, and the gene insertion was subsequently confirmed by polymerase chain reaction (PCR) and RE digestion. Finally, the integrity of the inserted gene fragment was validated by sequencing. In the second step, to generate the recMVA-BAC plasmid, the MEU gene fragment, along with the KanS fragment (containing the Kan cassette and the *I-Sce*I restriction site), was amplified from the pEP-MVAdVI-pH5-MEU plasmid using the MVA *Del*VI primers listed in Table 2. Following treatment with *Dpn*I, the purified PCR product was introduced into the MVA-BAC plasmid in *E. coli* GS1783, as previously described^34^. After removing the co-integrated Kan cassette from the inserted gene fragment, recMVA/MEU viruses were rescued using the purified recMVA/MEU BAC plasmid.

To rescue recMVA/MEU from the BAC plasmid, DF-1 cells at 85–90% confluency were transfected with the recMVA/MEU plasmid using Lipofectamin® 2000 transfection reagent (Thermo Scientific, USA). After 3 h, to reactivate MVA, cells were infected with the helper virus RFV at an MOI of 1 and gently rotated^34^. After 1 h, the wells were filled with RPMI medium supplemented with 10% FCS. Following incubation at 37 °C for 24–48 h, GFP expression in cells was checked using a CKK41 Fluorescence Microscope (Olympus, Germany). Following the successful rescue of viral progeny from the BAC plasmid, blind passages were performed repeatedly to eliminate the plasmid backbone and GFP cassette from the viral genome and clear RFV from the culture, which was verified by PCR using RFV-specific primers listed in Table 2. After confirming the integrity of the inserted gene fragment in the rescued recombinant viruses via sequencing, viral particles were propagated in DF-1 cells, purified on a 36% sucrose cushion, and titrated by calculating TCID_50_ in BHK-21 cells, following the method previously described by Kugler et al.^34^. The capability of recMVA/MEU to express the transgene (MEU antigen) in infected cells was verified by Western blotting using MEU-specific mouse antisera as the primary polyclonal antibody and HRP-conjugated goat anti-mouse as the secondary antibody.

### Mouse immunization and challenge

Female C57BL/6JRj mice (6–8 weeks old; Janvier, Le Genest-Saint-Isle, France) were used for immunogenicity and challenge studies. All animal experiments were conducted in accordance with protocols approved by the Government of Upper Bavaria (ROB-OB-55.2-2532.Vet_02-21-182) and complied with applicable laws and regulations governing animal welfare, health, consumer protection, and pharmaceutical use.

Mice were immunized intramuscularly with MEU-Flagellin protein (30 µg/dose) three times at one-week intervals. In a separate group, a heterologous prime-boost vaccination regimen was employed, featuring MEU-Flagellin protein (30 µg/dose) as the prime vaccination (administered intramuscularly twice), followed by a booster injection of the viral vector recMVA/MEU (3 × 10^7 virus particles) administered intramuscularly on the third injection. Mice injected with PBS or infected with *H. pylori* SS1 served as control groups. Mice were sacrificed 7 days after the final dose; sera and spleens were collected and analyzed for MEU-specific humoral and cellular immune responses via ELISA and intracellular cytokine staining (ICS).

To evaluate the efficiency of the multi-epitope vaccine candidate in preventing *H. pylori* infection, immunized mice were challenged two weeks after the final vaccination with three doses of *H. pylori* SS1 strain administered at a concentration of 1 × 10^9^ CFU, spaced 48 h apart. Mice were monitored regularly for changes in body weight and survival rates. Four weeks after the third inoculation, mice were sacrificed, and stomach tissue was collected to assess *H. pylori* colonization. Stomach tissues were washed with BPS, mechanically disrupted, and subjected to rapid urease testing, bacterial culture, and DNA extraction for PCR analysis targeting *H. pylori*’s *FliD* and *glmM* genes to confirm bacterial presence in the stomach.

To assess the therapeutic potential of the multi-epitope vaccine candidate, a chronic *H. pylori* infection model was first established in female C57BL/6 mice aged 6–8 weeks prior to immunization. To achieve this, mice were orally inoculated with *H. pylori* SS1 three times at intervals of 48 h, with each inoculation being administered at a bacterial dose of 1 × 10⁹ CFU. Four weeks post-infection, mice were randomly assigned to treatment groups: one group received three intramuscular administrations of the MEU-Flagellin protein (30 µg/dose) at one-week intervals. Another group was treated with a heterologous prime-boost regimen comprising two intramuscular doses of MEU-Flagellin (30 µg/dose), followed by a booster with recMVA/MEU (3 × 10⁷ viral particles). Control groups included untreated chronically infected mice and PBS-treated mice. Therapeutic efficacy was evaluated based on bacterial clearance rates and immune response activation. Two weeks after the final vaccination, stomach tissues were collected and processed according to the preventive study methodology to assess bacterial colonization via urease testing, bacterial culture, and PCR detection targeting *H. pylori*-specific genes. Additionally, cytokine production in spleen cells was assessed via ICS assay (see below for details) to evaluate vaccine-induced cellular immune responses in infected mice.

### Antibody titration by ELISA

MEU-specific antibody responses in mouse sera were determined using a quantitative ELISA assay. To this end, ELISA plates were coated with 1 µg/ml MEU protein for 4 h at RT. Two-fold serial dilutions of mouse IgG in 1 × PBS, starting at 250 ng/ml, were prepared to generate a standard curve and quantify IgG concentrations in the sera. After blocking the wells overnight at 4 °C with the SmartBlock^TM^ solution (CANDOR, Germany), a 1:100 dilution of the serum (in 1 × PBS) was added to the wells and incubated at RT for 1.5 h. The wells were washed five times with washing buffer (1 × PBS, 0.05% Tween 20) and then treated with HRP-conjugated anti-mouse secondary antibodies at RT for 1 h. Following five washing steps, the TMB substrate solution (Thermo Fisher Scientific, USA) was applied to the wells. After 5 min of incubation at RT in the dark, the reaction was stopped with 0.16 M H_2_SO_4_, and absorbance values were measured at 450 nm using an ELISA reader (Tecan, Germany). Finally, antibody titers were measured using an IgG standard curve.

### T-cell response evaluation by intracellular cytokine staining (ICS)

Splenocytes-associated lymphocytes were isolated from the spleens of vaccinated mice as previously described^22^. Isolated cells were stimulated overnight with MEU overlapping 15-mer peptides, along with 1 µg/ml Brefeldin A (Sigma-Aldrich, Germany). The cells were live/dead-stained with ethidium monoazide bromide (Invitrogen, USA). Pacific-Blue-conjugated anti-CD8α and PE-conjugated anti-CD4 antibodies (eBiosciences, Germany) were used for surface T-cell marker analysis. ICS was performed using FITC anti-IFNγ, PE-Cy7 anti-TNFα, and APC anti-IL-2 antibodies (eBiosciences, Germany) with the help of the Cytofix/Cytoperm kit (BD Biosciences, Germany). Flow Cytometry data were acquired using the CytoFLEX S (Beckman Coulter, USA) and analyzed using FlowJo software (Treestar, USA).

### Assessment of protection against H. pylori infection in vaccinated mice

To assess protection against *H. pylori* infection, bacterial cultures and PCR assays were employed using stomach samples collected from mice. Following euthanasia, stomach tissues were collected from mice under aseptic conditions. The stomach tissues were removed and immediately placed in sterile 1×PBS buffer to prevent degradation. Tissues were homogenized in 1×PBS buffer using a sterile homogenizer for subsequent bacterial culture and DNA extraction procedures. The homogenized stomach tissues were plated onto Columbia agar medium supplemented with 7% defibrinated sheep blood and *H. pylori*-selective antibiotics (Wüstner et al., 2017). The plates were incubated at 37 °C in a microaerophilic atmosphere (5% O₂, 10% CO₂, and 85% N₂) for 5 to 7 days. Colonies exhibiting typical *H. pylori* morphology were identified via the urease rapid test. Colony-forming units (CFU) were counted to quantify the bacterial load in the stomach tissue.

For PCR analysis, genomic DNA was extracted from homogenized stomach tissues using a DNA extraction kit (Qiagen, Germany) according to the manufacturer’s instructions. The extracted DNA was quantified using a Nanodrop 1000 (Thermo Fisher Scientific, Germany) to ensure adequate concentration and purity. PCR was performed using specific primers listed in Table 2 to detect the *H. pylori*-specific genes, *FliD* and *glmM*. The 50 µl PCR reaction contained 2 µl DNA template, 10 pmol of each primer, GoTaq Green Master Mix (Promega, USA), and nuclease-free water. PCR conditions comprised: 3 min pre-denaturation at 95 °C; followed by 35 cycles of 30 s denaturation at 95 °C, 30 s annealing at 60 °C, and 30 min extension at 60 °C; with a final 5-minute extension at 60 °C. Amplified products were visualized by electrophoresis in 1.5% agarose gel, stained with SYBR Safe Gel Stain (Thermo Fisher Scientific, USA), and the bands were observed under UV light. PCR results were evaluated based on the presence or absence of specific amplicon bands corresponding to the targeted *H. pylori* genes. The sensitivity and specificity of the culture and PCR methods were compared in assessing *H. pylori* infection.

### Statistical Analysis

Statistical significance of the data was determined using One-Way ANOVA to compare means between independent groups. All analyses were performed using GraphPad Prism software version 9.5.0, with results considered statistically significant at *p* < 0.05.

## Supplementary information

20260122_Supplementary Data

## Data Availability

All data supporting the findings of this study are available within the manuscript and the supplementary information.
