# Supplementary material for: A novel multi-epitope vaccine induces protective and therapeutic immunity against Helicobacter pylori
Source: NPJ Vaccines. 2026 Mar 16;11:69. doi: 10.1038/s41541-026-01409-9 (PMC13022432; doi:10.1038/s41541-026-01409-9)
Supplement: Supplementary file 1 — 20260122_Supplementary Data [file 41541_2026_1409_MOESM1_ESM.pdf]

Supplementary Data:

Figure S1: Molecular docking of MEU-flagellin and TLR5. This analysis identified potential binding sites between the fusion protein and the TLR5 receptor, demonstrating structural compatibility and providing valuable insights into the interaction between MEU-flagellin and TLR5.

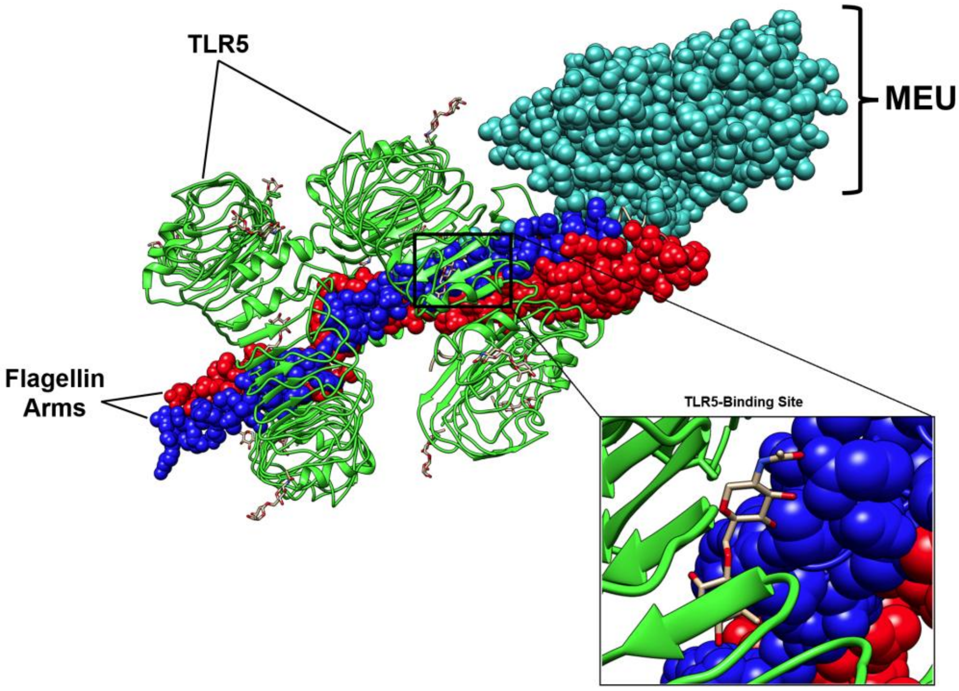

Figure S2: Purification of the MEU-Flagellin fusion protein. SDS-PAGE analysis of purified MEU-Flagellin fusion protein. The protein was expressed in *E. coli* BL21 (DE3) and purified using ion exchange chromatography (IEX) followed by size exclusion chromatography (SEC). A distinct band at the expected molecular weight of ~75.5 kDa confirms successful purification. (**A)** SDS-PAGE analysis of IEX fractions. Elution fractions containing the fusion protein were pooled, concentrated, and subjected to SEC for final purification. **(B)** SDS-PAGE analysis of SEC fractions confirming the purity of eluted MEU-Flagellin fusion protein. **(C)** Activation of the TLR5 signaling pathway by MEU-Flagellin fusion protein. TLR5 activation was evaluated using a SEAP reporter assay in HEK-Blue hTLR5 cells. The fusion protein elicited a dose-dependent increase in SEAP activity, reaching a plateau at 320–640 ng/mL, indicating saturation of TLR5 activation. Data represent the mean ± standard deviation (SD) from three independent experiments.

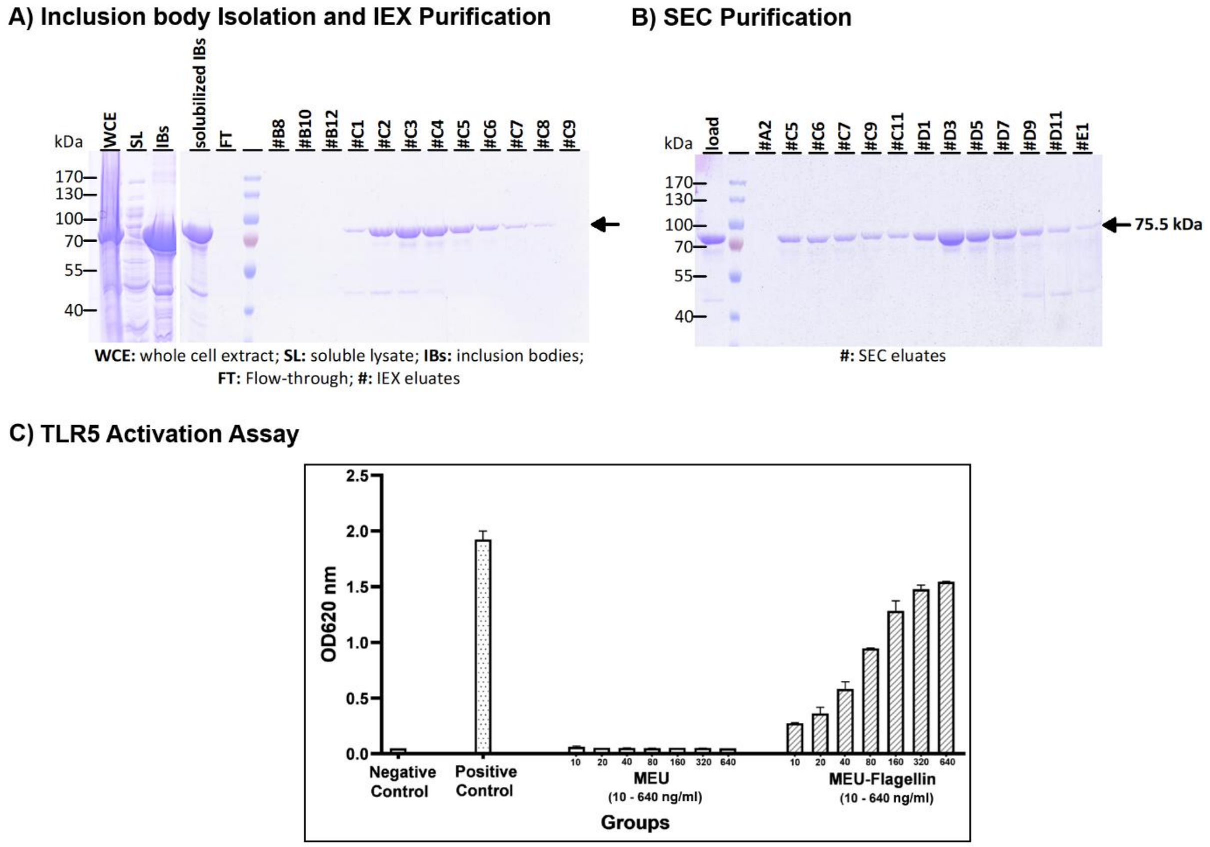

Original, uncropped images for Figure 3

(Cropped lanes are indicated by white lines in the gel images.)

1. PCR confirmation of recMVA vector construction following the first recombination step in *E. Coli*

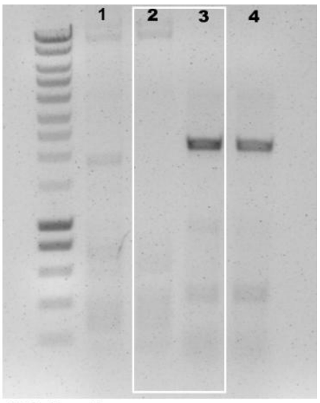

1&2: Negative
3&4: Positive

2. PCR confirmation: KanR cassette removal following the second recombination step in *E. coli*

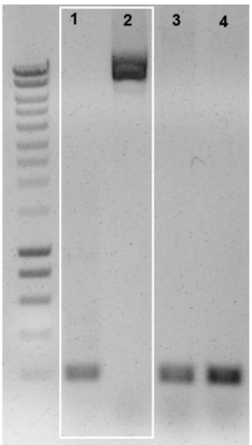

1,3,4: Negative for Kan removal
2: Positive for Kan removal

3. PCR confirmation of the presence of the MEU gene fragment in the genome of recombinant viral particles

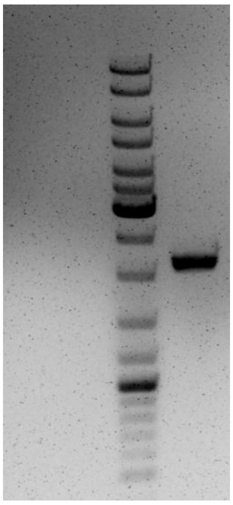

4. Original Western blot gel image

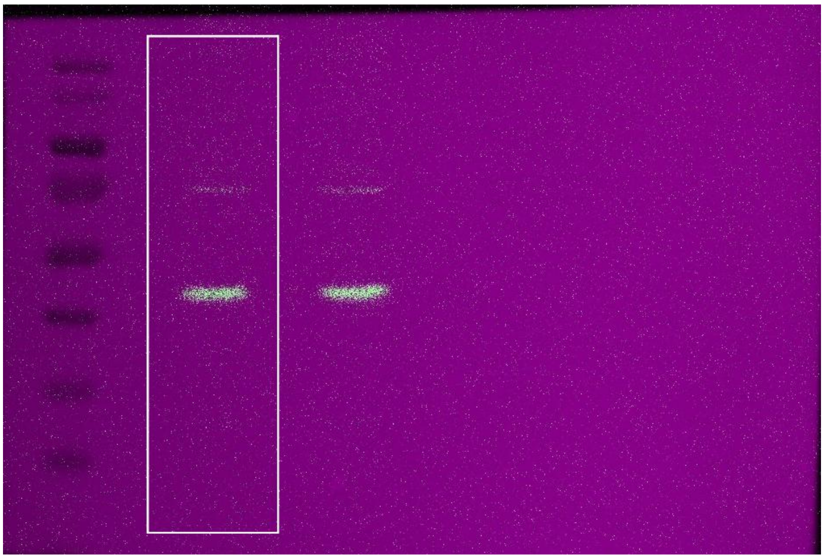

Original, uncropped images for Figure 5:

(Cropped lanes are indicated by white lines in the gel images.)

The original, uncropped PCR gel images contain results from two additional experimental groups, namely the Control-Adjuvant group and the 3× MEU-Flagellin group. These groups are not presented in the main figures of the manuscript for the following reasons. First, inclusion of the Control-Adjuvant group in the main dataset was avoided to prevent potential confusion in data interpretation, as this group did not contribute additional information relevant to the primary study objectives. Second, based on the immunogenicity data obtained in this study, as well as observations from our previous animal experiments, repeated immunization with the protein-based vaccine alone (3 × MEU-Flagellin) did not result in complete protection and showed only limited therapeutic efficacy. Consequently, this vaccination regimen was not advanced to the protection and therapeutic studies presented in this manuscript. The presence of these groups in the uncropped images reflects the experimental workflow and data transparency and does not affect the conclusions drawn from the presented results.

1. Evaluation of protective efficacy by PCR targeting the *fliD* and *glmM* genes

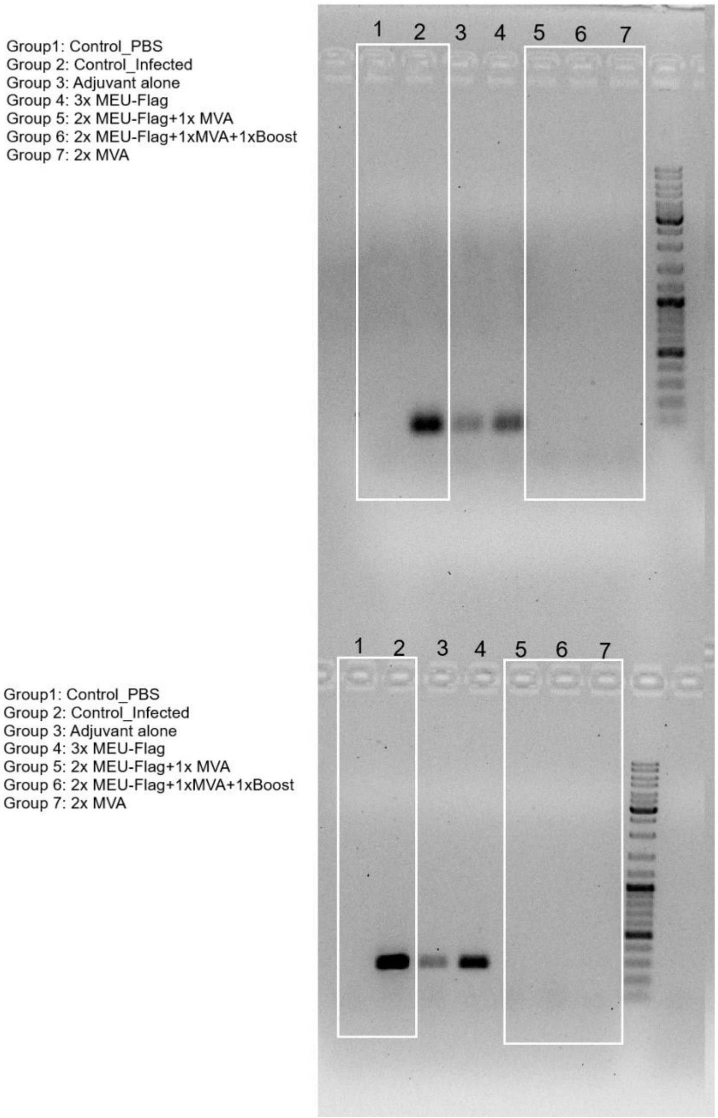

2. Evaluation of therapeutic efficacy by PCR targeting the *fliD* and *glmM* genes

Group1: Control_PBS
Group 2: Control_Infected
Group 3: Adjuvant alone
Group 4: 3x MEU-Flag
Group 5: 2x MEU-Flag+1x MVA
Group 6: 2x MEU-Flag+1xMVA+1xBoost
Group 7: 2x MVA

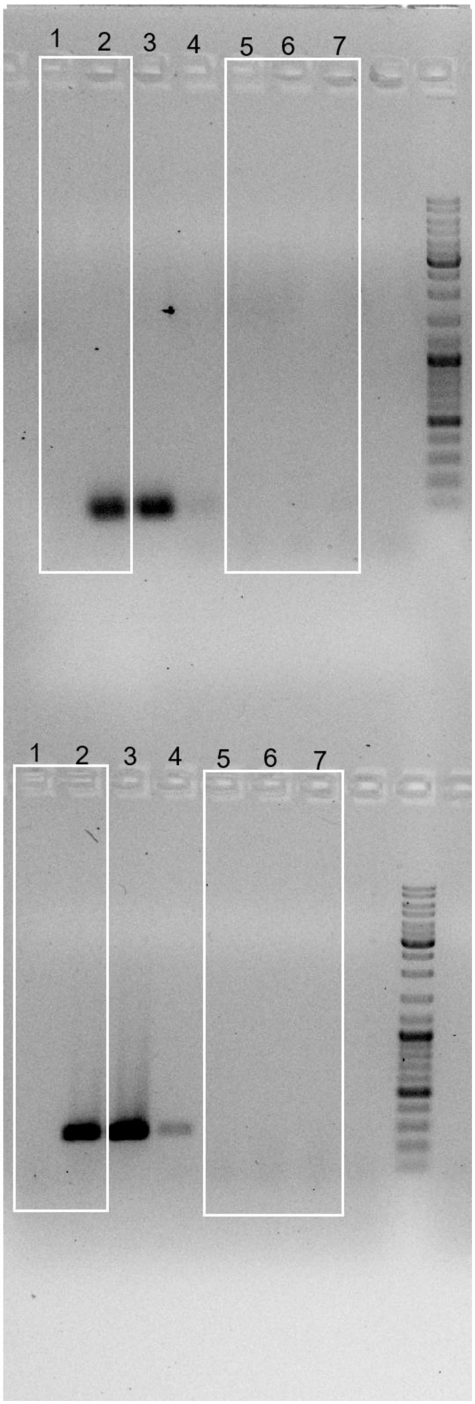

Group1: Control_PBS
Group 2: Control_Infected
Group 3: Adjuvant alone
Group 4: 3x MEU-Flag
Group 5: 2x MEU-Flag+1x MVA
Group 6: 2x MEU-Flag+1xMVA+1xBoost
Group 7: 2x MVA
